# Profile of patients and physiotherapy patterns in intensive care units in public hospitals in Zimbabwe: a descriptive cross-sectional study

**DOI:** 10.1186/s12871-015-0120-y

**Published:** 2015-10-07

**Authors:** Cathrine Tadyanemhandu, Shamila Manie

**Affiliations:** 1 Department of Rehabilitation, College of Health Sciences, University of Zimbabwe, PO Box AV 178., Avondale, Harare, Zimbabwe; 2 Division of Physiotherapy, Department of Health and Rehabilitation Sciences, Faculty of Health Sciences, University of Cape Town, Anzio Road, Observatory, Cape Town, South Africa

**Keywords:** Intensive care, Physiotherapy, Evidence-based practice, Profile of patients, Low-income countries

## Abstract

**Background:**

Physiotherapy is integral to patient management in the Intensive Care Unit. The precise role that physiotherapists play in the critical care differs significantly worldwide. The aim of the study was to describe the profile of patients and the current patterns of physiotherapy services delivered for patients admitted in the five public hospital intensive care units in Zimbabwe.

**Methods:**

A prospective record review was performed and records of all consecutive patients admitted into the five units during a two months period were included in the analysis. The data was collected using a checklist and the following were recorded for each patient: 1) demographic information, 2) admission diagnoses, 3) surgery classification, 4) method and time of mechanical ventilation 5) physiotherapy techniques and frequency and 6) the length of stay.

**Results:**

A total of 137 patients were admitted to five units during the study. The mean age of patients in the study was 36.0 years (SD = 16.6). A mortality rate of 17.5 % was observed with most of the patients being below the age of 45 years. The majority of the patients, 61(45 %) had undergone emergency surgery and were in the ICU for postoperative treatment, whilst only 19(14 %) were in the units for clinical treatment (non-surgical). On admission, 72(52.6 %) of the patients were on mechanical ventilation. The mean duration on mechanical ventilation for patients was 4.0 days (SD =2.7) and a length of stay in the unit of 4.5 days (SD = 3.0).

Of the patients who were admitted into the ICU 120 (87.6 %) had at least one session of physiotherapy treatment during their stay. The mean number of days physiotherapy treatment was received was 3.71 (SD = 3.14) days. The most commonly used physiotherapy techniques were active assisted limb movements (66.4 %), deep breathing exercises (65.0 %) and forced expiratory techniques (65.0 %).

**Conclusion:**

A young population admitted in the ICU for post-surgical treatment was observed across all hospital ICUs. The techniques which were executed in Zimbabwean ICUs showed that the goal of the physiotherapy treatment was mainly to prevent and treat respiratory complications and a culture of promoting bed rest still existed.

**Trial registration:**

PACTR201408000829202

## Background

The burden of critical care remains especially high in low-income countries due to critical care being in its infancy and also the presence of endemic diseases like HIV, tuberculosis and malaria [1]. Access to critical care resources in low-income countries is poorly described and there is little published data on the intensive care units (ICU) characteristics, unit capacity and the profile of patients in this setting [2]. Management of ICU patients requires significant resources which range from human to financial and the availability of the resources has direct impact on the outcome of patients admitted [2]. Often the nature of the critical illness and the modalities which are used to manage the prevailing condition can result in patients experiencing prolonged bed rest. This puts ICU patients at increased risk of developing complications [3, 4]. Baker [1] highlighted that there is also lack of training and awareness of the principles of critical care management in low-income countries. This said, some of the basic critical care, which will result in positive impact on patient care, does not need to be resource intensive. Firth and Ttendo [5] reported that implementation of systemic changes with a goal to effectively treat critically ill patients might result in a broader public health value.

Physiotherapy is integral to patient management in the ICU and current research is directed towards the acute respiratory management of patients [6]. It is also important to note that the precise role that physiotherapists play in the ICU differs significantly from one unit to the next, depending on factors such as the country in which the ICU is located, local tradition, staffing levels, training and expertise [7]. Physiotherapists have been urged to recognize and understand the serious complications secondary to ICU admission and to intervene at an early stage of the ICU stay to limit the consequences of these complications [8]. As critical care advances and ICU mortality declines, it is important to understand the complications that are experienced during admission which later influence long-term outcomes, so that evidence-based practice can be implemented in order to minimize them [9]. These complications include development of atelectasis, ventilator associated pneumonia, respiratory muscle weakness and deconditioning.

Respiratory ailments still remain a major cause of morbidity and mortality in lCU and chest physiotherapy is an important adjuvant in the treatment of most respiratory illness [10]. Chest physiotherapy is typically given to patients who are ventilated mechanically and those not, to aid the clearance of retained pulmonary secretions, to recruit collapsed distal lung units and to optimize the matching of ventilation and perfusion [11, 12]. The following techniques have been reported to constitute chest physiotherapy in high-income and medium-income countries ICU; airway suctioning, chest percussions, vibrations, positioning, mobilisation, manual hyperinflation, breathing exercises, use of assistive devices (PEEP bottle) and thoracic mobility exercises [7, 10, 13, 14].

Although a considerable amount of literature is available regarding physiotherapy management in ICU in most medium and high-income countries, relatively little has been written about physiotherapy in low-income countries in terms of availability, frequency and patterns. The aim of this study was to

1. To establish the profile of patients admitted into the five central hospital intensive care units in Zimbabwe over a period of 2 months in terms of age groups, gender and type of condition across all hospitals.
2. To determine the nature of the current physiotherapy services being offered in terms of the most frequently utilized methods of intervention and the frequency of service provision.

## Methods

A prospective record review was performed over a period of 2 months (June –August 2012) in five government hospitals in Zimbabwe. The five hospitals are the only quaternary level government hospitals with an ICU in Zimbabwe. They are all spread over the two cities with provincial status, which are Harare and Bulawayo. The hospital bed capacity for the five hospitals ranges from the smallest with 500 beds to 1800 beds for the largest referral hospital located in the Harare Province. The ICU occupancy rate for the hospitals varies according to the location and number of beds available in each unit, ranging from four to six beds. The ratio of hospital beds to ICU beds from the largest are as follows (300;1; 240:1, 250:1, 163:1 and lastly 125:1 for the smallest hospital). Some of the units have a separate adult and pediatric units whilst in others, adults and pediatrics are admitted in the same unit. The hospitals were given numbers to ensure anonymity of the institution.

The anaesthetists are in charge of the units and responsible for admitting and discharging the patients from ICU. A team of four doctors from the Anaesthetics department is always on cover in each hospital’s ICU at any given time, and that includes the consultant, registrar, senior health officer and senior resident medical officer. The ICUs are typically staffed with either one or two physiotherapists every day of the week as part of their clinical rotation. The physiotherapists usually cover the unit from 0800 to 1100 h am then they offer their afternoon session of physiotherapy from 1400 to 1600 h. An after-hours and weekend service is offered by one physiotherapist who will be on call on a rotational basis. The physiotherapy services in ICU independently assess the need for therapy for each patient on a daily basis. In all the hospitals, the physiotherapists do not require a physician or anaesthetist consultation or referral to initiate physiotherapy for the patients.

The study was approved by the ethics committee of University of Cape Town, (HREC; 190/2012), Zimbabwe’s ethics committee, (MRCZ/B/342) and Joint Research Ethics Committee (JREC/179/12) and the Institutional Review Board of each hospital. Records of all consecutive patients admitted into the five units during a two months period (03 June 2012 to 02 August 2012) with any diagnosis and from the age of 18 and older were screened for eligibility. The records of those who had died were also included. Records of patients found in the unit who had been admitted before the proposed date for data collection but had been receiving physiotherapy services were excluded from the study. A total of 150 patients were admitted across the ICUs during the research period, of which 137 patients met the inclusion criteria and provided consent. All patients provided written informed consent or had their next of kin consent on their behalf to participate in the study.

The sample size required to establish the prevalence of a health condition which was anticipated to be present in 10 % of the sample, with a precision of 2.5 % and a design effect of one and five clusters was 125, at 80 % confidence level (Epi Info Version 7.110). This was based on a population of 250, the number of patients who were estimated to be admitted over a period of two months.

Data was collected by five research assistants who were senior physiotherapists (more than 2 years of experience), based at each hospital but not working in the ICU during the rotation. The research assistants visited each unit at midday (12 pm) each day and accessed the patient’s notes and charts in order to complete the self-designed data extraction form (SDEF). The research assistants visited each bed daily and followed up the patients until either discharge from the unit or death whilst still admitted in the unit. The self-designed data extraction form consisted of three sections namely: 1). the demographic profile of the patient including age and gender, 2). concerned medical history which included the diagnosis of the patient, the location before ICU admission, the cause of condition and ventilator settings. The ventilator settings included all the possible ventilator modes and all the other parameters which are set on a ventilator. The subheadings under diagnosis, location before ICU admission and cause of condition were drawn up using the Ministry of Health and Child Welfare, Rehabilitation log book. The second section also consisted of factors documented in the literature that have an influence on patient short and long-term outcomes, namely duration on mechanical ventilation, length of stay in the unit and Glasgow Coma Scale [15, 16].

The last part consisted of the physiotherapy services. The specific physiotherapy techniques which were included in the self-designed extraction form were drawn from three studies done by Stiller [7]; Zeppos et al. [16]; Gosselink et al. [17]. The authors described and defined the current evidence-based physiotherapy practice in ICU. The list was used to identify the different treatment techniques being performed by the Zimbabwean physiotherapists. Continuous rotational therapy and electrical stimulation were not included in the list because of absent of the equipment needed to execute the techniques. The reasons why physiotherapy was not received by other patients were drawn up from the study done by Hanekom & Faure [13] in a South African ICU.

Data was analysed using Statistica (Version 11) and SPSS Statistical package Version 21. Central limit theorem, which states that given a statistically large sample (n >30), the shape of the sampling distribution will become more and more like a normal distribution, irrespective of the parent population [18] was applied in analysis and hence parametric statistics were used. The central tendency was described in terms of mean and variation of data as standard deviations (SD).

T-tests were used to show the mean difference in age, duration on mechanical ventilation and length of stay between males and females or between discharged and deceased patients. In order to do multiple comparisons ANOVA and Tukey test were performed.

ANOVA was used to indicate any difference across groups in age, duration on mechanical ventilation and length of stay. A Tukey test was used as the post hoc test to show differences within groups. Chi-square was used to show any difference in categorical data which included age groups, referring department, cause of condition and type of mechanical ventilation. Values were accepted as statistically significant at the 5 % level (*p* <0.05).

## Results

A total of 137 patients were drawn from five hospitals (Table 1) of which 80 (58.4 %) were females. The mean age of the sample was 36.0 years (*SD* = 16.6, range 18–83). Most of the patients were below the age of 45 years, 107(78.1 %) with only 8(5.8 %) patients above 70 years age group. Most of the patients in the unit had been referred from general surgery 52(38 %), followed by obstetrics and gynaecology 35(25.5 %). Only 6(4.4 %) of the patients had been referred to the unit from the Orthopaedics department. There was an association between the city and the referring department, (Chi-square = 21.4, *df* = 5, *p* = .001). Most of the patients from Harare, 47(47 %) were admitted into the ICU following general surgery and those from Bulawayo were admitted because of obstetrics and gynaecology, 14(37.8 %). Concerning the nature of admissions, 61(45 %) were under emergency surgery, 29(21 %) for elective surgery, 28(20 %) for traumatic surgery and 19(14 %) were on clinical treatment (non-surgical). Laparotomy for gastrointestinal (GI) disorders was the largest subgroup of the emergency surgery in 28(20.4 %) patients, with obstetrics and gynaecology being the next in 25(18.2 %) patients. Under non-surgical patients, 7(5.1 %) of the patients had neurological diseases and 6(4.4 %) had respiratory diseases.

**Table 1:** Demographic data of patients (*n* = 137) across hospitals

Characteristics		Hospital 1	Hospital 2	Hospital 3	Hospital 4	Hospital 5
		*n* (%)	*n* (%)	*n* (%)	*n* (%)	*n* (%)
Sample size		28 (20.4)	25 (18.2)	14 (10.2)	47 (34.3)	23 (16.8)
Age	mean (SD)	42.2 (18.9)	34.4 (18.3)	27.4 (7.3)	36.1 (15.4)	34.7 (16.4)
Sex	Male	12 (43)	9 (36)	2 (21)	28 (60)	6 (26)
	Female	16 (57)	16 (64)	12 (79)	19 (40)	17 (74)
Referring department	Cardiorespiratory	0	1 (4.0)	3 (21.4)	8 (17.0)	2 (8.7)
	General surgery	13 (46,4)	13 (52.0)	2 (14.3)	21 (44.7)	3 (13.0)
	Medical	7 (25.0)	2 (8.0)	0	4 (8.5)	4 (17.4)
	Neurological	0	0	1 (7.1)	9 (19.1)	3 (13.0)
	Obstetrics & gynaecology	7 (25.0)	9 (36.0)	8 (57.1)	5 (10.6)	6 (26.1)
	Orthopaedics	1 (3.6)	0	0	0	5 (21.7)
Location before admission	Another hospital	1 (3.6)	0	5 (35.7)	8 (17.0)	3 (13.0)
	Casualty	6 (21.4)	0	0	4 (8.5)	6 (26.1)
	General ward	7 (25.0)	5 (20.0)	2 (14.3)	4 (8.5)	3 (13)
	Labour ward	3 (10.7)	0	0	2 (4.3)	2 (8.7)
	Operating theatre	11 (39.3)	20 (80.0)	7 (50.0)	29 (61.7)	9 (39.1)

The most common cause for admission to ICU in 52(38.0 %) of the patients was as a result of an infection. The other causes were as follows, obstetrical complications present in 33(24.1 %), trauma in 28(20.4 %) and lastly neoplasm in 24(17. 5 %) of the patients. In relation to city, the majority of the patients in Harare the cause for admission was infection for the 46(46 %) of the patients, whilst patients from Bulawayo were in the unit secondary to obstetrical complications 14(38 %) and trauma 12(32 %). On admission, 72(52.6 %) of the patients were intubated of which 35(61.4 %) of the males were intubated compared to 37(46.3 %) of the females. The number of patients who died in ICU in this cohort was 24(17.5 %) with most of the patients in the age category < 45 years old. The majority of patients, 12(50 %) who died were in the unit following emergency surgery and only 3(13 %) patients were on clinical treatment.

As shown in Table 2, the mean days on mechanical ventilation for the patients who were discharged was 4.0 days (SD = 2.7, range 1–13) and for those who deceased it was 6.1 days (SD = 4.4, range 1–16). There was a significant difference in duration on mechanical ventilation between discharged males (mean = 4.73, SD = 3.29) and females (mean = 3.16, SD = 1.72) (*t* = 2.12, *df* = 49, *p* = .04).

**Table 2:** Mean duration period on mechanical ventilation in days for discharged and deceased patients based on location and referring department (*n* = 72)

	Discharged patients	Deceased patients
Duration period on mechanical ventilation (mean; SD)	4.0 (2.7)	6.1 (4.4)
Location before ICU admission (mean; SD)		
General ward	4.3 (1.2)	7.8 (6.1)
Casualty	3.2 (1.6)	9.0 (2.8)
Operating theatre	3.1 (1.8)	4.3 (4.1)
Labour ward	2	6.5 (3.5)
Another Hospital	7.7 (3.8)	6.3 (3.9)
Referring Department (mean; SD)		
General surgery	3.2 (1.9)	3.9 (2.5)
Medical	4.0 (2.2)	12.7 (2.9)
Obstetrics and Gynaecology	2.9 (1.6)	7.8 (5.2)
Orthopaedics	3.5 (0.7)	
Cardiothoracic	5.6 (3.8)	2.0 (1.4)
Neurological	8.6 (3.3)	5.3 (2.1)

An analysis of variance revealed a significant difference in mean days on mechanical ventilation in patients who were discharged and came from different locations before admission, F(4,46) = 7.759, *p* = .001 (Table 2). A post hoc Tukey test indicated a difference between the patients from another hospital and operating theatre (*p* = .001). Patients who had been referred by the neurosurgery department stayed on the mechanical ventilator for longer periods. ANOVA showed that there was a significant difference in the mean days on mechanical ventilation for discharged patients, depending on the referring department concerned, F(5,45) = 5.949, *p* = 001. A post hoc Tukey test showed significant difference between the mean days of patients who had been referred to the unit by the following departments: general surgery and medical (*p* = .02) and neurological and general surgery (*p* = .03).

The mean length of stay of the 113 patients who were discharged from the unit at the end of the study period was 4.5 days (SD = 3.0, range 1–16), compared to the LOS of the patients who died, which was 6.2 days (SD = 4.2, range 1–16) (Table 3).

**Table 3:** Mean length of stay in days depending on referring department/condition and location before ICU admission (*n* = 137)

	Discharged patients	Deceased patients
Mean length of stay in ICU (mean; SD)	4.5 (3.0)	6.2 (4.2)
Location before ICU admission (mean; SD)		
General ward	3.8 (2.4)	8.4 (5.3)
Casualty	4.6 (1.8)	7.0 (4.0)
Operating theatre	4.1 (2.5)	4.7 (4.1)
Labour ward	2.8 (0.8)	6.5 (3.5)
Another Hospital	8.4 (4.6)	6.3 (3.9)
Referring Department (mean; SD)		
General surgery	4.2 (2.6)	4.6 (2.3)
Medical	4.5 (2.5)	12.7 (2.9)
Obstetrics and Gynaecology	3.7 (2.2)	8.0 (5.4)
Orthopaedics	5.6 (1.1)	3.0
Cardiothoracic	5.5 (4.0)	3.5 (2.5)
Neurological	7.7 (5.3)	5.3 (2.1)

An ANOVA indicated that there was a statistically significant difference between mean length of stay of patients who were discharged and location before admission, F (4108) =8.025, *p* = .001, with patients referred from another hospitals staying longer followed by patients from casualty department. Looking at the mean LOS of patients from the different departments, patients who had neurological problems were found to have a higher mean LOS (mean = 6.9, SD = 4.5, range 2–16 days) as compared to the obstetrics and gynaecological patients (mean = 4.2, SD = 2.5, range 1–10 days) (Table 3).

Of the 137 patients who were admitted during this study period, 17(12.4 %) did not receive any physiotherapy treatment during their stay whilst 120(87.6 %) of the patients had at least one session of physiotherapy treatment. The majority of patients received physiotherapy treatment for 1–7days, 107(78.1 %) and only 13(9.5 %) received physiotherapy treatment for more than 8 days. The mean number of days physiotherapy sessions were received by the patients was 3.71 (SD = 3.14, range 0–15). The physiotherapy treatment was offered twice daily and the mean number of physiotherapy treatments received by each patient was 2.06 (SD = 1.62, range 0–8).

On consistency of the physiotherapy treatments, out of 120(87. 6 %) of the patients who had at least one session of physiotherapy treatment, only 32(23.4 %) patients received therapy throughout their ICU stay. The reasons why patients did not receive physiotherapy throughout their stay were; therapist did not come to the unit, 50(36.5 %); patient was admitted after hours, 21(15.3 %); patient had gone for theatre, 16(11.7 %); cardiovascular instability, 13(9.5 %) and patient was sedated, 2(1.5 %).

The most commonly used physiotherapy techniques in ICU were recorded and are reported in Table 4. The most frequently used techniques were active assisted limb movements in 91(66.4 %) of the patients whilst deep breathing exercises and forced expiratory techniques were executed in 89(65.0 %) of the patients. The frequency of techniques varied according to the category of surgery/condition. Patients who had undergone elective surgery had forced expiratory techniques, 22(75.9 %) and deep breathing exercises, 21(72.4 %) being executed more often compared to other techniques. For the trauma patients frequently executed techniques were passive limb movements, 23(82.1 %); vibrations, 24(85.7 %); percussions, 23(82.1 %) and suctioning 22(78.6 %). Emergency surgery patients received active assisted limb movements, 40(65.6 %) and deep breathing exercises, 38(62.3 %) more often compared to the other techniques.

**Table 4:** Physiotherapy techniques used in ICU

Technique	n	Frequency (%)
Active assisted movements	91	66.4
Deep breathing exercises	89	65.0
Forced expiratory technique	89	65.0
Circulation exercises	84	61.3
Vibrations	80	58.4
Suctioning	77	56.2
Percussions	77	56.2
Passive movements	71	51.8
Directed positioning	70	51.1
Breathing control	44	32.1
Application of oxygen	30	21.9
Active cycle of breathing techniques	27	19.7
Sit over edge of bed	24	17.5
Shaking	15	10.9
Active transfers	13	9.5
Insertion of a mouthpiece	12	8.8
Manual hyperinflation	2	1.5

The physiotherapy techniques executed also varied according to the mode of ventilation. For the patients who were on the volume controlled ventilation; suctioning, 34(24.8 %); percussions, 34(24.8 %); vibrations, 34(24.8 %) and passive movements of the limbs, 32(23.3 %) were mostly executed. Active assisted movements, 31(22.6 %), deep breathing exercises, 29(21.1 %) and forced expiratory techniques, 29(21.1 %) were mainly executed in patients who were on a face mask.

## Discussion

The mean age of the patients was comparable to other studies done in African countries which showed a younger population being admitted to the ICUs compared to the high-income countries [13, 19–22]. In medium and high-income countries, the mean age of patients admitted in the ICUs showed a much older population of above 60 years of age [23, 24]. The reason for this young population found in ICUs in developing countries has been reported to be due to different factors which include, a strict inclusion criteria into the ICU due to resources availability [13], life expectancy comparably low to that of developed countries and also the burden of diseases such as malaria, trauma, tuberculosis and HIV [21]. The UNAIDS report on the global AIDS pandemic (2012) highlighted that globally 34.0 million people were living with HIV by the end of 2011 and Sub-Saharan Africa remains most severely affected, with nearly 1 in every 20 adults (4.9 %) living with HIV. It has been reported that an estimated 0.8 % of adults aged 15–49 years worldwide are living with HIV [25]. Our study did not record the HIV status of all the patients who were enrolled because HIV testing was not routinely performed. Testing was only performed when treating physicians or surgeons had requested it.

Most of the ICUs in Zimbabwe are mixed medical and surgical units and there are no stand-alone units. Infection and obstetrical complications were the commonest causes of admission into the units in our study. Our results concur with literature which states that infection, trauma, post-operative treatment and perinatal complications are much more common causes of admission to an ICU in low-income countries than complications of chronic cardiac, vascular and pulmonary diseases which are prevalent in high to medium-income countries [22, 26–28]. The most performed surgery in our study was abdominal surgery, with laparotomy for gastrointestinal disorders being the common diagnosis. These findings were the same as found in Nigeria which showed laparotomy, followed by cardiothoracic surgery, were the commonest type of surgeries performed on the patients [19, 29]. The common cause of the gastrointestinal disorders was due to an infection, followed by neoplastic, and patients presented with intestinal obstructions, perforations and duodenal ulcers.

Our results showed that overall, patients stayed on mechanical ventilators for a short period. Comparing the duration on mechanical ventilation with literature, it showed that patients from low-income countries stayed on mechanical ventilation for a shorter period of time than in high-income countries [30–32]. The shortage of equipment and also the high demand for ICU beds might be the reason why patients have a shorter stay on mechanical ventilation in low-income countries [21, 33]. This same scenario has been supported by Mathivha [34] in South Africa who reported that due to extensive shortage of ICU beds especially in the public sector, intensivists have drawn up very strict admission, discharge and exclusion criteria to the units in order to be able to offer services to patients most likely to benefit from it. The factors which were found to affect duration period on mechanical ventilator and length of stay in the unit were location before ICU admission, category of surgery/condition and also referring department/condition and this was consistent with what was found in other studies [35, 36].

The frequency and the type of physiotherapy techniques have been reported to significantly vary based on the type of hospital and also the clinical scenario [37]. The results of our study indicated that 12.4 % of the patients did not receive any physiotherapy at all during their stay in ICU. Additionally, out of the 87.6 % who had physiotherapy, not all of them had routine physiotherapy every day. These results are similar to what has been found in other countries. In a study done in Australia to identify the availability of physiotherapy services, the results showed that a third of respondents stated that patients did not receive routine chest physiotherapy [10]. Also, another point was brought out of frequency of physiotherapy depending on the primary condition or diagnosis [37]. In the study by Hodgin et al. [37], frequency of physiotherapy was highest (87 %) in status post cerebrovascular accident and lowest (64 %) in chronic obstructive disease. Routine physiotherapy was more likely to be provided in patients who had neurologic problems than those who had medical conditions. The results of our study showed that patients who had obstetrics and gynaecology were more likely to receive routine physiotherapy compared to all the other conditions. The reason why physiotherapists mostly attended to patients from obstetrics and gynaecology was not clear. The above results show the variability in physiotherapy delivery across nations and the frequency might be due to the availability of staff or severity of conditions or complications.

Various reasons have been cited why patients did not receive physiotherapy routinely. The reason why most of the patients did not receive routine physiotherapy in our study was due to the absence of a physiotherapist. In most ICUs, there was only one physiotherapist covering the unit so if the physiotherapist did not report for duty and no other plan was made then the unit was not covered by a physiotherapist on that day. Also when patients were admitted after the physiotherapists scheduled ward visit, they did not receive therapy until the following day. These reasons differed from a South African study by Hanekom & Faure [13] who reported that reasons for non-treatment were due to patient instability, no physiotherapy indicated and patient not co-operative. The reasons of no therapist available to offer service or patient admitted after hours were not reported in South Africa. This might be due to the difference in staffing levels in these two countries.

The most commonly used techniques by Zimbabwean physiotherapists include active assisted movements, deep breathing exercises, forced expiratory techniques, circulatory exercises, vibrations, percussions and suctioning. From the techniques which were commonly used, the study showed that physiotherapy in Zimbabwe is more centred on the management of respiratory complications, which is the traditional focus of physiotherapy [14]. Similarly, in a study done in one unit in South Africa, the physiotherapists were found to use similar techniques with no mobilisation practices being implemented [13].

As physiotherapists’ awareness of the knowledge of the long-term complications has increased, this has led to the change in the standard clinical practice with the involvement of mobilisation as the treatment option [38]. This is evident in studies conducted in high-income and medium-income countries were most physiotherapists involve mobilisation as their part of intervention [14, 37, 39, 40]. These findings show that most nations have moved from the traditional focus of respiratory physiotherapy management alone and now involve mobilisation of patients as the standard practice. From the results of our study, it is clear that Zimbabwean physiotherapists have yet to make this shift in focus by including mobilisation as part of their intervention. Patients who received a number of different physiotherapy techniques were the obstetrics and gynaecology and general surgery patients. It showed that postoperative patients got more physiotherapy services than medical patients, which was similar to what Hodgin et al. [37] reported about United States physiotherapists. The physiotherapy management in Zimbabwe is more centered on prevention and management of postoperative pulmonary complications and this was why the majority of the patients who received the services were from obstetrics and gynaecology and those from general surgery.

The findings indicated the variability of physiotherapy across countries as stated by one author [7]. Not only was there a variability in the type of techniques but it has been noted that frequency of the services also varies. Norrenberg & Vincent [39] stated that the involvement of physiotherapists in more specialised techniques is also a function of the number of physiotherapists working exclusively in an ICU and this might be the reason of the variability found in all nations.

### Limitations and recommendations

The cross-sectional nature of this study makes it difficult to determine cause and effect relationships. Unfortunately, no severity scores were routinely taken in these intensive care units, therefore it was difficult to compare the severity of patients on admission. The physicians should consider using a validated scale such as APACHE score to measure the severity of illness on admission more objectively. Determining the complications which the patients presented with was not one of the objectives of this study, but some patients developed complications which made weaning them off the ventilator difficult and resulted in longer stays on ventilation, often for more than 13 days. Hence the need for further studies to be done looking at the development of complications in these patients and effect on patient’s outcomes.

## Conclusions

The results showed that a much younger population was admitted in the ICUs in Zimbabwe and most of the physiotherapy treatment done in these patients was to address primarily respiratory problems and not functional ones. The gender distribution differed within hospitals, depending on the incidence of condition leading to critical illness. The majority of the patients in the units were admitted for post-surgical treatment following emergency surgery. Abdominal surgery was the most performed procedure with patients having laparotomy for either of the following reasons: duodenal ulcer, perforations or intestinal obstructions. The cause of condition was mainly due to infection. Obstetrical complication was the second cause of condition, with the majority presenting with postpartum hemorrhage and eclampsia.

The discharged patients in our study had a shorter duration on mechanical ventilation and length of stay in the unit. However, there was no form of mobilisation of patients out of bed before ICU discharge. The results showed that physiotherapists in Zimbabwe are still relying on chest physiotherapy as a treatment option in ICU and not considering functional activities as early as possible to improve patient outcomes.

## Abbreviations

SDEF: Self-designed extraction form
PEEP: Positive end-expiratory pressure
ICU: Intensive Care Unit
HREC: Health Research Ethics Committee
MRCZ: Medical Research Council of Zimbabwe
JREC: Joint Research Ethics Committee
ANOVA: Analysis of Variance

## References

[1] Baker T (2009). Critical care in low-income countries. Trop Med Int Health.

[2] Murthy S, Leligdowicz A, Adhikari NKJ. Intensive Care Unit Capacity in low-income countries. A Systematic Review. Plos One. 2015;10. 10.1371/journal.pone.0116949 PMC4305307 25617837

[3] Milbrandt EB (2007). One small step for man …. Crit Care Med.

[4] Ross AG, Morris PE (2010). Safety and barriers to care. Crit Care Nurse.

[5] Firth P, Ttendo S (2012). Intensive Care in low-income countries- a critical need. New Engl J Med.

[6] Denehy L, Berney S, Skinner E, Edbrooke L, Warrilow S, Hawthorne G, Morris ME (2008). Evaluation of exercise rehabilitation for survivors of intensive care: protocol for a single blind randomised controlled trial. Open Crit Care Med J.

[7] Stiller K (2000). Physiotherapy in Intensive Care. Chest.

[8] Pattanshetty RB, Gaude GS (2011). Critical illness myopathy and polyneuropathy - A challenge for physiotherapists in the intensive care units. Ind J Crit Care Med.

[9] Desai SV, Law TJ, Needham DM (2011). Long-term complications of critical care. Crit Care Med.

[10] Chaboyer W, Gass E, Foster M (2004). Patterns of chest physiotherapy in Australian Intensive Care Units. J Crit Care.

[11] Barker M, Adams S (2002). An evaluation of a single chest physiotherapy treatment on mechanically ventilated patients with acute lung injury. Physiother Res Int.

[12] Denehy L, Berney S (2006). Physiotherapy in the intensive care unit. Phys Ther Rev.

[13] Hanekom S, Faure MAC (2006). Outcome evaluation of a South African surgical ICU – a baseline study. S Afr J Crit Care.

[14] Berney S, Haines K, Denehy L (2012). Physiotherapy in critical care in Australia. Cardiopulmonary Phys Ther J.

[15] Berenholtz SM, Dorman T, Ngo K, Pronovost PJ (2002). Qualitative review of intensive care unit quality indicators. J Crit Care.

[16] Zeppos L, Patman S, Berney S, Adsett J, Bridson JM, Paratz JD (2007). Physiotherapy intervention in intensive care is safe: an observational study. Aust J Physiother.

[17] Gosselink R, Clerckx B, Robbeets C, Vanhullebusch T, Vanpee G, Segers J (2011). Physiotherapy in the Intensive Care Unit. Netherlands J Crit Care.

[18] Fagerland MW (2012). T-tests, non-parametric tests, and large studies — a paradox of statistical practice ?. Med Res Methodol.

[19] Isamade E, Yiltok S, Uba A, Isamade E, Daru P (2007). Intensive care unit admissions in the Jos University teaching hospital. Niger J Clin Pract.

[20] Manie S, Hanekom S, Faure M (2008). Profile and length of stay of coronary artery bypass graft patients in the Cape metropolitan area. S Afr J Crit Care.

[21] Kwizera A, Dünser M, Nakibuuka J (2012). National intensive care unit bed capacity and ICU patient characteristics in a low-income country. BMC Res Note.

[22] Sawe HR, Mfinanga JA, Lidenge SJ, Mpondo BCT, Msangi S, Lugazia E, et al. Disease patterns and clinical outcomes of patients admitted in intensive care units of tertiary referral hospitals of Tanzania. BMC Int Health Human Rights. 2014;14. 10.1186/1472-698X-14-26 PMC4204389 25245028

[23] De Freitas ERFS (2010). Profile and severity of the patients of intensive care units: prospective application of the APACHE II index. Rev Lat Am Enfermagem.

[24] Wunsch H, Angus DC, Harrison DA, Linde-Zwirble WT, Rowan KM (2011). Comparison of medical admissions to intensive care units in the United States and United Kingdom. Am J Respir Crit Care Med.

[25] World Health Organisation. UNAIDS report on the global AIDS epidemic. Joint United Nations Programme on HIV/AIDS 2012. UNAIDS/JC2417E.

[26] Adhikari NKJ, Fowler RA, Bhagwanjee S, Rubenfeld GD (2010). Critical care and the global burden of critical illness in adults. Lancet.

[27] Dünser MW, Baelani I, Ganbold L (2006). A review and analysis of intensive care medicine in the least developed countries. Crit Care Med.

[28] Riviello E, Letchford S, Achieng L, Newton M (2011). Critical care in resource-poor settings: Lessons learned and future directions*. Crit Care Med.

[29] Bolaji BO, Kolawole IK (2005). The Intensive Care Unit of the University Teaching Hospital, Ilorin, Nigeria: a ten year review (1991–2001). S Afr J Anaesth Analg.

[30] Malkoç M, Karadibak D, Yildirim Y (2009). The effect of physiotherapy on ventilatory dependency and the length of stay in an intensive care unit. Int J Rehabil Res.

[31] Rosenthal VD, Rodrigues C, Álvarez-Moreno C, Madani N, Mitrev Z, Ye G, Salomao R, Ulger F, Gaunche-Garcell H, Kanj SS, Cuellar LE, Higuera F, Mapp T, Femandez-Hidalgo R, INICC members (2012). Effectiveness of a multidimensional approach for prevention of ventilator-associated pneumonia in adult intensive care units from 14 developing countries of four continents: findings of the International Nosocomial Infection Control Consortium. Crit Care Med.

[32] Size M, Borstein E, Halsma HJ (2005). One-year audit of admissions to the intensive care unit of the queen Elizabeth Central Hospital, Blantyre. Malawi Med J.

[33] Towey RM, Ojara S (2007). Intensive care in the developing world. Anaesthesia.

[34] Mathivha LR (2002). ICUs worldwide: an overview of critical care medicine in South Africa. Crit Care.

[35] Parkhe M, Myles PS, Leach DS, Maclean AV (2002). Outcome of emergency department patients with delayed admission to an intensive care unit. Emerg Med.

[36] Goldhill DR, Mcnarry AF, Hadjianastassiou V, Tekkis P (2004). The longer patients are in hospital before Intensive Care admission the higher their mortality. Intensive Care Med.

[37] Hodgin KE, Nordon-Craft A, McFann KK, Mealer ML, Moss M (2009). Physical therapy utilization in intensive care units: results from a national survey. Crit Care Med.

[38] Skinner EH, Berney S, Warrillow S, Denehy L (2008). Rehabilitation and exercise prescription in Australian intensive care units. Physiotherapy.

[39] Norrenberg M, Vincent J-L (2000). A profile of European intensive care unit physiotherapists. Intensive Care Med.

[40] Kumar J, Maiya A, Pereira D (2007). Role of physiotherapists in intensive care units of India: a multicenter survey. Indian J Crit Care Med.

